# Stakeholders’ engagement in co-producing policy-relevant knowledge to facilitate employment for persons with developmental disabilities

**DOI:** 10.1186/s12961-020-00548-2

**Published:** 2020-04-17

**Authors:** Akram Khayatzadeh-Mahani, Krystle Wittevrongel, Lisa Petermann, Ian D. Graham, Jennifer D. Zwicker

**Affiliations:** 1 School of Public Policy, University of Calgary, Downtown Campus, 906 8th Avenue S.W., 5th Floor, Calgary, Alberta T2P 1H9 Canada; 2 Health Services Management Research Center, Institute for Futures Studies in Health, Kerman University of Medical Sciences, Kerman, Iran; 3 Libin Cardiovascular Institute of Alberta, Calgary, Canada; 4 Ottawa Hospital Research Institute, Ottawa, Ontario Canada; 5 School of Epidemiology and Public Health, Faculty of Medicine, University of Ottawa, Ottawa, Ontario Canada

**Keywords:** Integrated knowledge translation, Stakeholder, Engagement, Knowledge co-production, Research, Developmental disability, Nominal group technique

## Abstract

**Background:**

Persons with developmental disabilities (PWDD) face a number of individual, environmental and societal barriers when seeking employment. Integrated knowledge translation (IKT) involves ongoing and dynamic interactions between researchers and stakeholders for the purpose of engaging in mutually beneficial research to address these types of multi-faceted barriers. There is a knowledge gap in the IKT literature on effective stakeholder engagement strategies outside of the dissemination stage to inform policy. In this paper, we report on a number of engagement strategies employed over a 2-year period to engage a wide range of stakeholders in different stages of an IKT project that aimed to investigate the ‘wicked’ problem of employment for PWDD.

**Method:**

Our engagement plan included multiple linked strategies and was designed to ensure the meaningful engagement of, and knowledge co-production with, stakeholders. We held two participatory consensus-building stakeholder policy dialogue events to co-produce knowledge utilising the nominal group technique and the modified Delphi technique. A total of 31 and 49 stakeholders engaged in the first and second events, respectively, from six key stakeholder groups. Focused engagement strategies were employed to build on the stakeholder dialogues for knowledge mobilisation and included a focus group attended only by PWDD, a stakeholder workshop attended only by policy/decision-makers, a webinar attended by human resources professionals and employers, and a current affairs panel attended by the general public.

**Results:**

Our findings suggest that the level of engagement for each stakeholder group varies depending on the goal and need of the project. Our stakeholder dialogue findings highlight the inherent challenges in co-framing and knowledge co-production through the meaningful engagement of multiple stakeholders who hold different ideas and interests. Focused outreach is needed to foster relationships and trust for meaningful engagement.

**Conclusions:**

In addition to providing guidance on how to implement adaptable meaningful engagement strategies, these findings contribute to discussions on how IKT projects are planned and funded. More studies to explore effective mechanisms for engaging a wide range of stakeholders in IKT research are needed. More evidence of successful engagement strategies employed by researchers to achieve meaningful knowledge co-production is also key to advancing the discipline.

## Background

Canadians with developmental disabilities face lower employment rates (24%) than any other disability group in the country [1]. Despite rights-based legislation, poor employment rates suggest that persons with developmental disabilities (PWDD) experience pervasive barriers to participating in the labour force. Even some of existing programmes and policies act as a barrier to employment for PWDD. For instance, Assured Income for the Severely Handicapped (AISH) is a Government of Alberta programme that provides financial assistance to adults who have severe disabilities that significantly limit their ability to work. However, an unintended consequence of the programme is that it provides a disincentive for some PWDD to work additional hours, as they receive less after-tax earnings and transfers than those who work fewer hours at the same wage level [2]. Individual, environmental and societal factors all impact employment outcomes for PWDD [2, 3]. Policy-makers and decision-makers need to address prioritised barriers to employment for PWDD more holistically by designing policies considering employers and the workplace, persons with developmental disabilities, and the broader society [2]. This implies that the problem to be solved (i.e. low employment rates for PWDD) requires a cross-sectoral and multifaceted stakeholder approach [4].

A persistent gap exists in translating research findings to policy and practice [5, 6]. To reduce this know–do gap and to better use the research evidence, integrated knowledge translation (IKT) is a promising approach to ensure the co-production (also known as co-creation, co-generation and co-design) of policy-relevant knowledge for use in addressing multifaceted ‘wicked’ social policy problems like employment for PWDD [5, 7–9]. The term ‘wicked’ problem, first proposed by Rittel and Webber in 1973 [10], refers to complex social system problems that are ill-formulated and are continually evolving with many causal levels and no single solution that applies in all circumstances. IKT is well suited to address these problems as it strives to ensure that those impacted by science have a say in the discovery process and thereby increase the likelihood of shifting uptake. It helps align research directions to stakeholders needs and values [11] and holds the potential to enhance the relevance of research and facilitate uptake of its results [5, 12].

IKT involves an ongoing and dynamic relationship between researchers and stakeholders for the purpose of engaging in mutually beneficial research throughout the entire research process, from conceptualisation through implementation and evaluation, in order to co-produce knowledge relevant to policy and practice change [12–15]. IKT was first introduced by the Canadian Health Services Research Foundation in the late 1990s and early 2000s as a Knowledge Exchange concept [5, 16]. It was then adopted and refined by the Canadian Institutes of Health Research (CIHR) in the 2000s and was coined as IKT [5].

Stakeholders are often defined as groups who (1) are essential to the implementation of resulting policies, (2) have expert knowledge and (3) have an interest in the outcome of research [17]. In our research we viewed stakeholders as those who are interested in the evidence but may or may not be using the evidence in their decision-making. Empirical evidence around best practices and effective strategies for stakeholder engagement in research using an IKT approach is lacking [8, 18–20]. Stakeholder engagement may take place at different stages of a research project. Likewise, the degree or level of engagement as well as the role of different stakeholders can also vary depending on the goals and needs of the project [21]. Concerns have been expressed that some research engagement efforts are tokenistic, relying on stakeholders passively receiving information [22], and acting as a ‘weak public’ rather than a robustly and meaningfully engaged one [23, 24]. The literature on stakeholder engagement, especially public/citizen engagement, in research is highly diverse and theoretically heterogeneous [25]. Empirical evidence on how stakeholders’ inputs are integrated into the research process is also lacking [26]. While there have been efforts to present some frameworks and models of stakeholder engagement in research [18, 25], there is limited evaluation of these frameworks [19, 25–27] due, in part, to limited theoretical development to underpin evaluation tools [19, 26, 28]. In contrast to public and patient engagement literature that is rich in terms of clarifying the role and degree of stakeholders’ engagement by providing a number of stakeholder engagement taxonomies [29], the IKT literature has given minimal attention to the stakeholders’ role and their degree/level of engagement/participation in research [8]. There are taxonomies in the form of a hierarchy of stakeholder engagement characterising engagement as operating across a spectrum/continuum of volume with the upper end being co-production/partnership [30, 31]. In our research, we adopted the stakeholder engagement continuum presented in the CIHR Framework for Citizen Engagement [32]. This framework, adopted from the Health Canada framework [33], illustrates five levels of engagement, from low to high, across a continuum/spectrum, including (1) inform and educate (distribution of information to help stakeholders understand the issue/problem, options and solutions), (2) gather information (gathering stakeholders perspective and concerns), (3) discuss (two-way information exchange with stakeholders), (4) engage (in-depth deliberation) and (5) partner (joint decision-making). In our research, our goals and intent were to achieve the highest level of engagement (i.e. partnership) and to use a true IKT approach to inform policy for employment for PWDD.

In this paper, we report on our stakeholder engagement approach that included multi-pronged strategies employed over a 2-year period to engage a wide range of stakeholders in different stages of a project to investigate the ‘wicked’ social problem of employment for PWDD. Building on patient and public engagement literature, we describe how we engaged multiple stakeholders, how our engagement strategies worked, and how our engagement evolved/matured over time in terms of both engagement strategies and stakeholders’ responses to those strategies. We use our experiences to describe tactical engagement strategies as well as make strategic recommendations to improve the capacity of researchers in engaging multiple stakeholders toward a true IKT of partnership level engagement.

## Methods

**Fig. 1:**
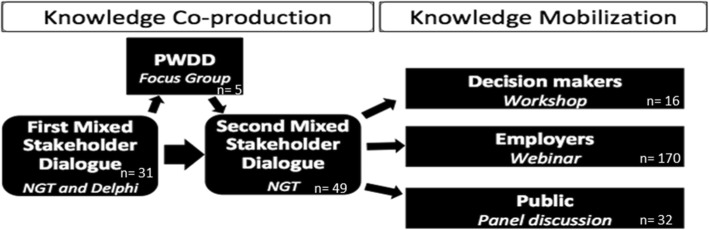
Overview of engagement strategies

We adopted a case study approach in order to better understand how to engage a wide range of stakeholders in different stages of research and what challenges and opportunities for engagement exist. We selected the case of employment for PWDD given the broad perspectives of stakeholders needing to be engaged in addressing this multifaceted ‘wicked’ problem [2]. Our engagement approach involved two phases. Phase one involved knowledge co-production in which we engaged a wide range of stakeholders in two stakeholder policy dialogue events and a focus group (FG) attended only by PWDD to deepen the findings of stakeholder dialogue. In phase two, knowledge mobilisation, we employed focused engagement strategies to reach key stakeholder groups and share with them the policy-relevant knowledge co-produced during our stakeholder dialogue events. These tailored engagement strategies included a workshop attended exclusively by policy/decision-makers, a webinar attended by human resources (HR) professionals and employers, and a current affairs panel attended by the general public (Fig. 1).

### Optimisation of stakeholder partnership strategies

#### Phase one: knowledge co-production

Knowledge co-production is defined as “*joint working between people or groups who have traditionally been separated into categories of user and producer*” [34]. It refers to equal participation in developing knowledge outputs. To achieve co-production, collaborative activities are needed in which researchers and other stakeholders work together to co-produce new knowledge to address particular policies or problems [35]. To co-produce knowledge, we partnered with multiple stakeholders in two stakeholder dialogues and used well-documented consensus-building methods such as the nominal group technique (NGT) and Delphi. We held a FG with PWDD following the first stakeholder dialogue event to correct for low representation and validate and contextualise the findings.

##### First stakeholder dialogue

A stakeholder dialogue is referred to an organised meeting of stakeholders that is structured to a greater or lesser extent by means of consensus-building techniques/methods [36]. Consensus is defined as a settlement that all stakeholders can live with and does not mean that all stakeholders are fully satisfied with [37]. In public policy literature, consensus-building is considered the key objective of stakeholder policy dialogues [37, 38]. By deploying consensus-building techniques, stakeholder policy dialogues aim to co-develop joint policy recommendations that meet the needs of all engaged stakeholders [37]. The attention to stakeholder dialogues has increased in recent years driven, in part, by (1) disappointment with the role of scientific knowledge alone in policy [39] and a greater appreciation for other stakeholders’ knowledge and expertise [40], (2) call for more transparent democracy to improve the legitimacy of policies and decisions [41], and (3) an increased number of educated and knowledgeable citizens in contemporary societies [42].

We convened the first stakeholder policy dialogue event to focus on identifying the barriers, and solutions to those barriers, to employment for PWDD, held in June 2017. We used purposeful, convenience and snowball sampling approaches to identify stakeholders who had experience in working with, employing or caring for individuals with developmental disabilities, or those with lived experience. We identified stakeholders who had an interest in our research findings, some of whom (e.g. PWDD) would not implement the research findings while others, such as employers or policy/decision-makers, could potentially implement the findings. Our inclusion criteria focused on individuals who were engaged in supporting, providing services to or developing policy for PWDD. Our exclusion criteria were individuals who could not provide full informed consent and whose place of residence was, and work focused beyond, the geographic and policy catchment region of Alberta, Canada. We shared a policy brief with all stakeholders prior to the stakeholder policy dialogue event, which provided a background understanding of existing policy supports, rights-based protection legislation and income support policies that are relevant to PWDD in Canada.

The stakeholder dialogue featured both presentations and facilitated discussions. It started with two plenary presentations, one by the research team and the other by a policy-maker. The research team presented the latest evidence on the state of the problem and the policy-maker presented current policies regarding the problem under consideration. As part of the stakeholder dialogue, we employed two participatory consensus-building methods – the NGT [43] and a modified Delphi technique [44]. The purpose of the NGT was to enable stakeholders to identify, explore and rank-order barriers to employment for PWDD. The purpose of the Delphi was to establish consensus around potential policy solutions to address the prioritised barriers. The modified three-step Delphi technique was initiated during the first event and completed online following the event.

##### Nominal Group Technique (NGT)

NGT was first introduced by Delbecq and Van de Ven [43] as a process to generate and prioritise ideas and enable equal participation of group members [45]. It is a stepwise, democratic and participatory consensus-building process that helps create a list of collectively established priorities [46]. It helps generate ideas in relation to both problems and policy solutions, which are then discussed and rank-ordered by all participants [45, 47]. NGT is particularly well adapted to our research because of its specific focus on empowering all participants [45, 46]. The facilitated discussions and voting process of the NGT minimised typical power dynamics of diverse stakeholder groups and allowed varied stakeholders to contribute equally to group discussions [48]. Five key advantages of NGT include (1) discouraging idea domination by more vocal or powerful members of the group [49], (2) group members’ satisfaction as it requires little preparation and allows for immediate dissemination of results [50], (3) limiting possible researchers’ biases as data are interpreted by participants [51, 52], (4) giving participants a sense of achievement as, by using this method, participants are more likely to reach clear outcomes [45], and (5) a money-, time- and resource-efficient technique as it generates a sizable amount of information within a short time frame using little resources in a single occasion [43, 48]. We employed an adapted version of the NGT that entails five steps (see Additional file 1 for a detailed description of each step taken during our first event), as follows: (1) silent generation, (2) round robin engagement, (3) clarification, (4) categorisation and (5) ranking [50]. We pre-assigned stakeholders to groups based on their stakeholder category/group identification (e.g. PWDD and caregivers, policy/decision-maker, employer, etc.). Each group was composed of a combination of stakeholders and contained 6–8 participants, which allowed for the representing of diversity and easy exchange of ideas [53]. Our NGT processes were facilitated by an internationally experienced professional facilitator, which was the key to consensus-building during the two policy dialogue events [54].

##### Modified Delphi

We used a modified three-step Delphi technique to identify and rank order policy solutions to overcome the identified and prioritised barriers to employment for PWDD as well as criteria to evaluate proposed policy solutions. Delphi is a qualitative research method employed to systematically incorporate stakeholders’ knowledge, opinions and expertise to establish an informed group consensus on a problem [55]. The Delphi technique has proven to be an effective tool to obtain consensus among diverse stakeholders based in different locations and of varying backgrounds [44]. The process aims to reach convergence of opinions and views where its key characteristics – stakeholder’s anonymity, iterative feedback and statistical group response – allow stakeholders to freely express their view and re-engage with that perspective based on receiving the group feedback [56]. Unlike NGT, the Delphi does not require research participants to interact directly with each other to establish consensus, but instead provides participants with an equal opportunity to provide their input and feedback anonymously [57]. Similar to NGT, the online Delphi process ensures that all individual and stakeholder groups have an equal voice in the outcome as they do not experience in-person interactions, which is important where stakeholders hold different interests and come from different institutions (we note that not all stakeholders have institutions, for example, in our case, PWDD and their family caregivers as well as non-profit sector participants) [58, 59]. The anonymity in the Delphi online process is useful to mitigate the influence of power relationships and to prevent the domination by a particular individual or stakeholder group [58]. We initiated an in-person brainstorming practice in the first stakeholder dialogue event followed by two rounds of online survey.

The first step of the Delphi technique (i.e. brainstorming) was completed in-person following the NGT, where stakeholders brainstormed potential policy solutions to tackle the identified and prioritised barriers. We then used these policy solutions to develop an online survey, the second step of the Delphi technique. In this survey, we asked stakeholders to rate the importance of each item on a five-point Likert scale ranging from strongly disagree (1), disagree (2), neutral (3), agree (4), and strongly agree (5). The survey was self-administered; thus, stakeholders were able to answer without the risk of response being influenced. We carried out standard descriptive statistical analysis using SPSS at the end of the first round of the online survey. The analysis was performed through aggregating individual scores rather than stakeholder groups. The prioritised policy solutions (those that received greater than 80% of participant agreement during the first round) were compiled and included in the second round of the online survey (the third step of the Delphi), which was initiated among the same stakeholders 4 weeks after first online survey. We asked stakeholders to prioritise and rank-order the top five policy solutions in terms of perceived importance in addressing existing barriers as well as top five criteria for evaluating the adequacy of policies in terms of perception of impact on employment accessibility and inclusion for PWDD. This iterative process allowed stakeholders to re-evaluate and re-consider their responses based on aggregated results.

##### Focus group with PWDD

Following the first stakeholder policy dialogue event, as we saw a low representation from PWDD, we held a FG with PWDD in November 2017 to understand their views on the prioritised barriers and solutions and determine if the identified priorities aligned with their views and lived experience. FG is a qualitative research method that employs group discussions to explore a particular issue/problem [55, 60]. In FG, a group of individuals who share common experiences are gathered by researchers to discuss the research topic from their personal experiences [59, 61, 62]. We considered FGs as the most appropriate method to better understand the views of PWDD on the prioritised barriers and solutions reached in the first stakeholder dialogue event. Our key goal of holding this FG was to deepen our understanding of the barriers with the workforce experience of PWDD as well as providing important context to the feasibility, applicability and suitability of policy solutions identified (see Khayatzadeh-Mahani et al. [2] for more details).

##### Second stakeholder dialogue

A second stakeholder policy dialogue was held in June 2018 and focused on (1) the development of employer-specific, action-oriented solutions to reduce barriers to employment for PWDD relative to the workplace, and (2) the exploration of options for enhancing employers’ knowledge, capacity, attitudes and management practices for employing PWDD. This event expanded on a top barrier identified and prioritised in the first dialogue – addressing employers’ knowledge, capacity, attitudes and management practices, and the prioritised solution – focused on promoting employer training and knowledge. For this stakeholder policy dialogue, we again used purposeful, convenience, and snowball sampling and relied on connections made with those vested in the area of developmental disabilities and employment from the first stakeholder dialogue event.

This stakeholder dialogue again featured both presentations and facilitated discussions. The layout was refined from that of the first event, with plenary presentations interspersed throughout the day and the showcasing of local and national leaders highlighted. An NGT was employed to prioritise policy solutions relative to the workplace. At the start of the event and before embarking on the NGT process, stakeholders were asked to individually record their answer to the following question: ‘what is the biggest challenge you see when hiring an individual with a developmental disability?’ Once everyone had recorded their answers, they were asked to stand up and move around the venue to find all of the other participants who had similar answers. A decision about whether or not the answer was similar was up to the participants. While they were sharing their answers, participants were also asked to introduce themselves to one another in an effort to cultivate a safe space for sharing throughout the day.

Following the NGT and during lunchtime, there was a keynote address on ‘breaking down barriers to inclusive workplaces’ in which two employers shared their best practices of employing PWDD with the audience. Thirteen employers attended solely this portion of the event. After the keynote, participants were asked to co-produce a plan for moving from the potential solutions to action. The purpose was to generate actionable solutions, including what the proposed actions are, who is responsible and when it should be completed by. This was designed to allow stakeholders to break down solutions into pieces that felt more manageable and could be implemented in a series of steps, the topic of the final session of the day by a Canadian public policy scholar who has made substantive contributions to the understanding of disability policy in Canada.

#### Phase two: knowledge mobilisation

Knowledge mobilisation is defined as a range of strategies that help move research findings into society and bring new ideas into the research setting. It also refers to the extent to which a stakeholder that needs knowledge for a specific issue can be effectively matched with those who possess that knowledge [63]. We deployed focused strategies, described below, to mobilise knowledge co-produced during stakeholder dialogues to specific stakeholders. Our broad vision for these knowledge mobilisation activities was to help shift the focus from the charitable model of hiring PWDD to making the business case for inclusion. Our goals for these focused engagement strategies were (1) to foster relationships and trust for meaningful engagement, (2) to educate and raise awareness among different stakeholders of our findings, and (3) to exchange and discuss our research findings with different stakeholder groups.

##### Ministry stakeholder workshop

To further engage decision-makers, results of our two stakeholder events and FG were shared with decision-makers in the Alberta Ministry of Community and Social Services via a workshop in December 2018. Prior to the workshop, participants were provided the results of two stakeholder dialogue events and FG, a School of Public Policy Communiqué [60] outlining the importance of the issue, and presentation slides tailored to their specific Ministry and mandates.

##### Webinar

To connect with employers and HR professionals, we held a webinar in February 2019 aimed at sharing findings and educating and raising their awareness on opportunities and challenges. It was a 45-min online presentation to employers and HR professionals across sectors, sharing findings from stakeholder dialogues, the FG and the workshop.

##### Current affairs panel

The current affairs panel is an open forum for discussing the latest public and social policies with the general public. The panel aimed at discussing the applicability of engagement findings in a broader context.

## Results

### Engagement at various phases of research

In contrast to other studies in which stakeholders have been mostly engaged at the initial phase of prioritising research questions or at the end of the research process (dissemination and implementation of research findings [15]) our stakeholders were engaged throughout the research process/continuum, including the stages of recruitment, data collection, data analysis and interpretation, and dissemination, with the aim of subsequent implementation of our research findings. Using snowball sampling, our stakeholders (e.g. community-based disability organisations) helped recruit participants for both stakeholder dialogue events. Our consensus-building methods (i.e. NGT and Delphi) allowed our stakeholders to participate in data collection, data analysis and interpretation. Following the two stakeholder dialogue events, we shared the findings with stakeholders to solicit feedback before greater dissemination via various means.

For an overview of our engagement activities, see Table 1 below.

**Table 1:** Overview of 2-year engagement activities

	Engagement activity	Time	Number of stakeholders engaged	Engagement goal	Engagement level	Challenges	Solutions
**Phase one: knowledge co-production**	First stakeholder dialogue	June 2017	Multiple stakeholders^a^ (31)	To collectively prioritise policy barriers and potential solutions to employment for PWDD	Partner	Low participation from PWDD	1) Visiting a third-party disability organisation to present first event findings to PWDD 2) Held a separate focus group solely with PWDD
	Focus group with PWDD	Nov 2017	PWDD (5)	To deepen our understanding of prioritised barriers and solutions	Gather information	Difficulty to get access to PWDD and limited trust	1) Working through a third-party disability organisation 2) Continued and sustained communication and trust building relationships
	Second stakeholder dialogue	June 2018	Multiple stakeholders^a^ (49)	To co-develop employer-specific, action-oriented solutions to reduce policy barriers to employment for PWDD	Partner	Low participation from policy/decision-makers	Held a separate workshop in their workplace (i.e. Ministry) tailored to their specific stakeholder group
**Phase two: knowledge mobilisation**	Ministry stakeholder workshop	Dec 2018	Policy/decision-makers (16)	To provide space for dialogue about research findings with a focus on practical implementation of findings	Discuss	1) Buy-in on research findings 2) Limited time and difficulty to access 3) Reinterpretation of findings	1) Visiting stakeholders in their workplace 2) Sharing tailored information in advance of workshop
	Webinar	Feb 2019	HR professionals, employers (170)	To educate and raise awareness among employers of our findings	Inform or educate	1) Lack of in-person interaction between participants 2) Distractions by other activity in office/location 3) Difficult to gauge level of engagement with content	Incentives and professional credits attached to participation via continuing education by the HR professional organisation partnership
	Current affairs panel	Feb 2019	General public (32)	To discuss the applicability of engagement findings in a broader context	Discuss	1) Varied participant backgrounds and knowledge base 2) Asymmetry of information among participants	Including subject experts in the panel with both tacit and explicit knowledge to provide differing connection point for participants

*HR* human resources, *PWDD* people with developmental disabilities

^a^These include PWDD and their family members/caregivers, employers, non-profit organisations and other disability-serving organisations, vocational training professionals, and researchers and academics

#### Results of first stakeholder policy dialogue

With the first stakeholder dialogue we were able to involve 31 stakeholders from 6 distinct stakeholder groups, as follows: (1) PWDD and their families/caregivers (*n* = 5), (2) employers from medium-sized private businesses and third sector employers (*n* = 3), (3) non-profit organisations and other disability serving organisations (*n* = 5), (4) decision-makers and policy-makers who were engaged in policy design and programmes mainly from Alberta Ministry of Community and Social Services and the Premier’s Council on the Status of Persons with Disabilities (*n* = 5), (5) vocational training professionals (*n* = 3), and (6) researchers and academics (*n* = 10). Overall, 55% of our participants were female and 45% were male. As a result of the first stakeholder dialogue NGT, the participating stakeholders were able to prioritise barriers to employment for PWDD. Stakeholders reached consensus on the top three barriers, including (1) employers’ knowledge, capacity, attitudes and management practices, (2) late start to the ‘concept of work’ and workplace culture education, and (3) stigma. This prioritisation exercise revealed that employers’ knowledge and attitude is the key barrier/problem, which guided the focus of our second event to explore employer-specific, action-oriented solutions. During the NGT, described above and elaborated in Additional file 1, our diverse stakeholders co-framed the problem (i.e. barriers to employment for PWDD), which was reflected in collective priorities.

The systematic consensus-building process of Delphi allowed our stakeholders to identify 28 policy solutions, which shaped the content of the first online survey [2]. A total of 15 policy solutions received more than 80% of stakeholders’ votes during the first round of the online survey. We included these policy solutions in the final round of the Delphi (the second round of the online survey), whereby the top five policy solutions were equally endorsed and prioritised by all stakeholder groups; these were the following: (1) promoting employer training and knowledge, (2) promoting better education (building employability and job skills into education) in high school to enable smooth transition to post-secondary or employment, (3) changing AISH to remove barriers and disincentive to work, (4) increasing employment opportunities, and (5) education on inclusion, acceptance and human difference to be taught early on in both school and the workplace.

#### Results of FG with PWDD

Since we had low participation from PWDD in our first stakeholder policy dialogue (*N* = 1), we engaged only PWDD (and not their families/caregivers as before) in a FG. We recruited five PWDD (3 females and 2 males) through a third-party disability organisation. The research team was invited to present findings from the first stakeholder dialogue event to a group of clients of this organisation, which helped build trust and access to FG participants. Our FG participants not only expressed their views on NGT and Delphi results, but they developed their arguments and negotiated the issues in question. This engagement resulted in providing important context to, and a better understanding of, both barriers and policy solutions.

Following the first stakeholder dialogue event and FG with PWDD, we published a Communiqué with a target audience of employers and policy/decision-makers [60] as well as an original article in a peer-reviewed disability-related journal [2].

#### Results of second stakeholder policy dialogue

We were able to engage 49 stakeholders in the full-day stakeholder dialogue event, including (1) PWDD (*n* = 6), (2) employers (*n* = 10), (3) non-profit organisations and other disability serving organisations (*n* = 15), (4) vocational training professionals (*n* = 5), and (6) researchers and academics (*n* = 13). Overall, 59% of our participants were female (*n* = 29) and 41% (*n* = 20) were male. While there was confirmed attendance from a number of decision-makers and policy-makers, due to circumstances beyond our control, none were present for the event. It is important to note that many of the attendees wore multiple hats, although only their primary stakeholder group was included in this analysis. Additionally, there was a 1-hour keynote address on breaking down barriers to inclusive workplaces that drew an additional 13 employers solely for this portion of the day. Employers expressed that they wanted to learn but were not comfortable engaging in deeper discussion regarding policy solutions.

During the second event, which focused on actionable policy solutions, our stakeholders co-produced policy solutions, again using NGT. Before embarking on NGT and in response to the exercise run at the outset of the event, to create space for stakeholders to know each other, five themes arose from the answers provided during this exercise:

1. *Match* – ‘is the individual a good fit for the role and the company?’
2. *Entry* – ‘the traditional interview process (e.g. behavioural based) and question types are especially challenging for people with developmental disabilities.’
3. *Support* – ‘employers need for supplemental training’, ‘individuals with developmental disabilities may need support when they first start in the role’, and ‘support to get off AISH’.
4. *Capacity* – ‘can individuals with developmental disabilities actually do the job?
5. *Stigma* – ‘we see the disability first and the possibility second’ and ‘general lack of understanding about developmental disabilities’.

The event then employed an NGT technique to leverage the expertise and experiences of diverse stakeholders with varied talents, knowledge and skills to build consensus-based priorities around actionable policy solutions. Inspired by stakeholders’ own experiences as well as the keynote address from an employer, participants were given the opportunity to develop as many potential solutions as possible for one of the five themes described above. This exercise resulted in 121 options of which 10 received priority (Table 2).

**Table 2:** Top ten actionable policy solutions

Theme	Policy solution
Match	1. Well-defined role definition
	2. Creating awareness and training about disabilities
Entry	1. More functional interviews – task based, exercise driven, as opposed to question based
	3. Alternative ways to ‘sell yourself’/showcase in interviews beyond traditional models
Support	1. Segregation of health benefits and finance benefits within Assured Income for the Severely Handicapped (AISH)
	2. Task analysis and consultation with accommodation specialist
Capacity	1. Adopt culture that works toward individual success through flexible expectations; risk-fail culture, reinforce understanding and celebrate ‘failure’
	2. Define job/role in quantifiable clear terms based on operational experience not job posting ‘wish list’ in order to set real required standard for job performance and productivity management
Stigma	1. Changing perceptions of what disability means, for example, information specific to disability, changing language and understanding
	2. ‘Get past charity and give people opportunities’ and ‘Do the right thing – see beyond the stereotype’

#### Results of Ministry stakeholder workshop

A total of 16 participants attended the workshop, where the majority (*n* = 13) were female. Our objective at the workshop was to provide space for dialogue and discussions about the prioritised barriers and policy solutions with a focus on implementation of the evidence co-produced during our stakeholder engagement efforts. While our intention was to provide policy/decision-makers with conceptual opportunities and how other stakeholder groups understood the problem and policy solutions, we were aware of the potential challenges of reinterpretation [61]. However, we did not face this challenge and were met with interest and active participation in discussions during, and following, the research team presentation and workshop.

#### Results of webinar

The webinar was attended remotely by 170 HR professionals and employers. Some questions from the material presented were raised and discussed and the slides and materials discussed were shared with participants. Questions were curated by a moderator, so direct interaction with participants was not facilitated, and impeded the ability for dialogue. Consistent with the well-documented literature on disadvantages of distance and web-based learning/e-learning, including webinar, we faced a number of challenges for this engagement activity. These include (1) lack of face-to-face interaction with participants, (2) limited likelihood of asking a question in online discussion/dialogue, (3) distractions by other activities in immediate vicinity, (4) lack of time to participate, (5) problems with Internet access, and (6) difficulty in gauging level of engagement with content [62, 64]. Professional credits were associated with continuing education through the HR professional organisation partnership. To maintain status as an HR expert, HR professionals must complete a specified number of professional development activities, which incentivised participation.

#### Results of current affairs panel

The panel was attended by 32 members of the general public. Findings from the engagement activities were shared and a panel discussion featured two employer ‘champions’ with experience hiring PWDD, and a vocational training expert who discussed the applicability of our engagement findings in the employer context. The key challenge expected to be associated with this engagement activity was the varied participant backgrounds and knowledge base, where some participants had never heard of the topic before. The research team anticipated this challenge and, to best mitigate it, the panel included two employers, a vocational training professional, and a researcher, all of whom have extensive knowledge or experience in this area. Having a range of voices with both tacit and explicit knowledge provided differing connection points for participants.

### Engagement challenges

Our 2-year engagement experience shows how complicated, time-consuming and resource-intensive the engagement process is. The research team spent a considerable amount of time to apply for and modify research ethics to accommodate the ebb and flow of new ideas as well as to communicate and build relationships with a wide range of stakeholders through various communication channels. We communicated with stakeholders through in-person interaction at their organisations, invitations to visit the research institute, and followed up through telephone and email. These iterative and ongoing communications over a long period of time and in secure and safe places (e.g. stakeholders’ organisations or research institute) allowed questions, contemplation and iterative information exchange that together helped generate a common language and a greater mutual understanding [65, 66]. There were also significant financial expenses associated with our engagement activities, mostly logistic expenses (e.g. meals, venue rental, travel expenses, etc.). We also noticed most of our stakeholders, particularly employers and policy/decision-makers, work within traditional institutional structures (e.g. ever-increasing specialisation and fragmentation, siloed service delivery) that are not conducive to engagement in research as well as increased demands on time and resources.

Another challenge we identified in our co-production phase (i.e. stakeholder policy dialogues) was the informational asymmetry that was present throughout the dialogues. In one extreme, policy/decision-makers were more knowledgeable about the logistics associated with policy recommendations and, on the other extreme, employers lacked the fundamental knowledge about challenges faced by PWDD seeking employment, to allow them to engage in solution-oriented discussion. We identified the need for further input from both policy/decision-makers and employers in response to priority strategies identified in the dialogues, through more focused engagement strategies and in forums these stakeholder groups were better situated to engage in.

### Stakeholders’ evaluation of engagement

Analysis of post-event evaluation forms revealed stakeholders’ satisfaction with the use of tacit knowledge during the two policy dialogue events. Non-academic stakeholders appreciated that their experiences and knowledge (i.e. tacit knowledge) were respected and integrated to co-produce knowledge. Analysis of these evaluation forms revealed that stakeholders, most specifically PWDD and their families, felt an integral part of the process, valued and motivated to partner with us. Stakeholders also appreciated the role of our highly experienced facilitator and the use of structured qualitative methodologies in giving them a safe space to speak and to be heard.

## Discussion

In this study we presented our observations of diverse engagement activities in which we used principles of IKT to actively and meaningfully engage a wide range of stakeholders at various stages of our research to co-produce and mobilise policy-relevant knowledge. We engaged different stakeholders at various levels of engagement depending on our research needs and goals. Our knowledge co-production phase (stakeholder policy dialogues and FG) engaged stakeholders at partnership level while our knowledge mobilisation phase (stakeholder workshop, webinar and current affairs panel) engaged stakeholders at lower levels of engagement, including informing, gathering information and discussing (Table 1). Our findings make a contribution to IKT research as limited literature exists on how to engage a wide range of stakeholders in various stages of research, especially in developing policies to support PWDD [67]. There is limited evidence of employing an IKT approach in disability research [68]. Most of the previous research on employment for PWDD has been driven by survey or population data e.g. [1, 69–75] or from the perspective of a single stakeholder group [70, 76–79].

Our findings further contribute to the literature on how to engage PWDD in research along with other stakeholder groups. PWDD were not presented in the research context until 1970, when their engagement in research was mostly limited to being tested, counted, observed, analysed and pathologised [80, 81]. They were never asked for their views until the movement in the early 1990s that saw a shift from a medical model of disability towards a social model [81, 82]. At this time, there was a strong recognition that PWDD are not only reliable research participants with the rights to express their valid opinion but the best authority on their experiences and views [82]. Although PWDD were actively engaged throughout our research process, we highlight the importance of utilising multiple strategies to more meaningfully engage this population group in research and address the diverse lived experiences of stakeholders.

Over 2 years of engagement activities we learned two key principles for good practice, including (1) knowing well who your key stakeholder groups are as the beginning part of the engagement process, especially awareness and understanding of diversity in the ideas, interests and institutional context of different stakeholders, and (2) trust building and ongoing interactions between researchers and stakeholders. To facilitate this, next time we would start the problem definition (conceptualisation) phase with engagement of each stakeholder group separately through techniques such as in-depth interviews or FGs before embarking on multi-stakeholder engagement strategies such as a stakeholder policy dialogue. This will help better understand stakeholders (values, interests, context) and help develop trust. This approach also allows stakeholders the opportunity to provide semi-structured feedback and experiences before more structured consensus-building techniques such as NGT and Delphi techniques are used.

### IKT challenges: managing different ideas, interests and institutions

Our findings suggest caution in the use of consensus-building techniques, such as NGT and Delphi, in collective framing or co-framing of a problem and co-production of policy solutions by multiple stakeholders who hold different ideas, interests and institutions. While the NGT and Delphi were good approaches to start with, it was important to engage PWDD and policy/decision-makers outside of these dialogues to ensure the priorities aligned with their needs, values and perspectives. It is well documented that ideological conflicts between stakeholders who represent the interests of diverse institutions is a key factor to failure of most collaborative efforts [83, 84]. The social construction of a problem by different stakeholders, the types of people/organisations that benefit or lose from a policy solution, and the impact size of a potential policy all influence its adoption and subsequent policy change [85, 86]. In addressing a multifaceted ‘wicked’ problem like employment for PWDD, different stakeholders define and frame the problem differently. This affects the potential policy options each stakeholder group envisions for solving the problem [4, 87] and showcases the importance of further engagement strategies for defining a ‘wicked’ problem and its policy solutions [88]. Co-framing of a problem and policy solutions by multiple stakeholders has been shown to be one of the keys to successful collaborations [87, 89]. Using consensus-building methods during our stakeholder policy dialogues, our multiple stakeholders co-framed barriers and co-produced policy solutions in a holistic way that span through (1) PWDD and their families/caregivers, (2) employers and (3) society.

We engaged a wide range of stakeholders – PWDD and their families/caregivers, non-profit organisations and other disability serving organisations, decision-makers and policy-makers, vocational training professionals, and researchers and academics – who hold different ideas and interests, and come from different institutions. Parsons [90] defines ideas as “*claims about descriptions of the world, causal relationships, or the normative legitimacy of certain actions*”. Ideas are also defined as discourses, arguments and evidence advocated by stakeholders. Ideas help construct the problems and issues that reach the political agenda [91]. They impact how different stakeholders define or frame a problem and how they perceive different policy options to be feasible, acceptable and effective [92]. Use of the NGT in our first stakeholder dialogue event revealed the top three barriers to employment for PWDD that reflect both external (environmental or societal) and internal (personal) factors, including (1) employers’ knowledge, capacity, attitudes and management practices (external), (2) late start to the ‘concept of work’ and workplace culture education (internal or personal) and (3) stigma (external or societal) [2]. It is interesting that different stakeholders used different sources of evidence/knowledge, including explicit and tacit knowledge [93], in their discussions during step three of NGT (i.e. clarification). For instance, while researchers mostly shared explicit knowledge (e.g. survey results) with other stakeholders, PWDD shared their lived experiences and employers shared their experience of employing or working with PWDD – both tacit knowledge. This is how, we argue, co-learning took place during our two stakeholder dialogue events. Both explicit and tacit knowledge merged during the ranking step of NGTs, resulting in the co-framing of barriers to employment during the first event NGT and in co-production of action-oriented policy solutions during the second event NGT. In other words, the multi-causal barriers reflect collective ideas of, and problem co-framing by, multiple stakeholders. In the same vein, the prioritised policy solutions targeting employers, PWDD and broader society reflect the collective ideas of, and normative legitimacy given to, policy options by diverse stakeholders.

Interests refer to the groups and individuals who stand to gain or lose from a policy change and power relationship among them [91, 94]. Policy change often follows changes in the configuration of interests and power [95]. Power depends predominantly on resources, but also on the ability to gain visibility, establish coalitions, encourage sympathy for certain social problems, and persuade the public and media [96]. To reduce the power gap between different stakeholders in our engagement events, we used the NGT and Delphi techniques, both of which have proven to ensure different voices are given equal weights [45, 48]. Despite this, we held a separate FG with PWDD to ensure our engagement with this group was meaningful. The two most distinctive interest groups in our engagement events were those of employers and PWDD. While employers were more concerned about the cost of accommodation and perceived loss of profit and productivity, PWDD mostly argued for more inclusion and better accommodation in the workplace. Notwithstanding the different interests of these two groups as well as the interests of other stakeholders, the NGT and Delphi processes contributed to collective framing of the problem and co-production of policy solutions.

Institutions play a significant role in shaping the behaviour and policy choices of stakeholders [97]. Stakeholders’ actions are influenced by their institutional contexts, which define how stakeholders think about what they are doing, both individually and collectively; this constrains or incentivises the choices and policy options/solutions available [98]. In our engagement events, the use of the specific qualitative methodologies was our strategy to minimise the impact of institutional context as stakeholders from diverse institutional settings such as public, private and non-profit organisations were brought together. Institutions also refer to present and past policies and government structures that influence the development of new policies [91]. In this way, institutions structure the policy through influencing how new ideas come to the surface and how they are expressed in government decisions [91]. The ‘old historical institutionalists’ believe that institutions sustain ‘path dependence’, slow down change and ensure policies remain the same [99]. This group uses institutions to account for policy stability. Path dependence explains how a country’s constitution and past policies influence subsequent political dynamics. As we move toward implementation of our research findings, we hope that meaningfully engaging different stakeholders would overcome the persistent phenomenon of path dependence, which explains why policy change and innovation is very difficult in the Canadian system [100, 101].

The emphasis on ‘problem solving’ rather than ‘problem definition’ or ‘problem structuring’ [102] in the case of ‘wicked’ problems is arguably one of the key reasons for policy failure as it assumes that all stakeholders are aware of other stakeholders’ position as well as their own [103, 104]. This finding has implications for funding/granting agencies as the research funding models in some funding agencies (except funders who promote solutions-based research using an IKT approach) tend to push researchers to solve problems they think exist instead of allowing stakeholders to co-define and co-frame problems and co-produce solutions through meaningful engagement. The co-framing and co-production of knowledge allow stakeholders to move toward higher levels of engagement or co-production/partnership. As we move towards the implementation of our research findings, we hope this co-framing of the problem and co-production of policy solutions, in a way that considers multiple stakeholders who hold different ideas, interests and institutions, helps the adoption of our research findings in both policy and practice.

### Maturity in engagement

An interesting finding of our two stakeholder policy dialogue events was the maturity in terms of stakeholders increasing their level of engagement toward co-production/partnership overtime. The engagement strategy was designed for co-production but, in the first event, stakeholders were not ready to embrace it until they developed trust. By the second event, participants were more ready for co-production. We observed more willingness and a higher rate of participation from all stakeholders in our second event (aside from policy/decision-makers), perhaps due to snowball sampling and word-of-mouth of stakeholders from the first event. For instance, we had only one PWDD participating in our first event (with families/caregivers acting as proxies in a few additional cases), but in the second event we had six participants (and additional family members present who did not act as proxies). Here, we argue for the importance of embedded champions from each stakeholder group who attended our first policy dialogue event and encouraged engagement from their peers in the second one.

There were pragmatic and intrinsic challenges associated with developing maturity over time. Our findings illustrate the difficulty of engaging multiple stakeholders for complex wicked problems and the significant amount of time and resources required to establish effective communication strategies, continuous interactions, relationships and trust for a meaningful engagement. Iterative communications with stakeholders help promote listening and hearing of ideas and motivates collaboration [65]. Our findings support other emerging evidence that suggests that, while IKT has positive impacts [5, 6, 8, 105], this approach has not been widely adopted and practiced [5, 8] and researchers tend to employ traditional methods of conducting and disseminating research (e.g. end of grant knowledge translation) [106, 107]. There are barriers that render attempts to use an IKT approach unsuccessful, including (1) lack of incentives for researchers and stakeholders to engage in the costly and prolonged process of knowledge co-production, (2) lack of capacity to address challenges intrinsic to coordinating complex prolonged partnerships between researchers and stakeholders [8, 108], (3) a variety of perspectives, values and interests that different stakeholders hold and pursue [7, 109], and (4) limited researchers’ knowledge about effective engagement strategies [8, 110].

Many research funding agencies around the world, such as CIHR in Canada, INVOLVE (a project established by the British National Institute of Health Research to increase public engagement) in the United Kingdom, and the Patient-Centered Outcomes Research Institute (PCORI) and Quality Enhancement Research Initiative (QUERI), both in the United States, are now encouraging partnership between researchers and stakeholders through funding opportunities. In Alberta, the home jurisdiction for the case study at the centre of this paper, Alberta Innovates – a provincial research funding agency – has introduced an IKT approach named Partnership for Research and Health Innovation in the Health System and the Collaborative Research and Innovation Opportunities programme to promote partnership between researchers and stakeholders. The CIHR Strategy for Patient-Oriented Research (SPOR) is another funding partnership that strives to engage patients and their families/caregivers as partners in the research process to ensure that research is focused on patient-identified priorities leading to better patient outcomes and better health policies [111]. Our findings have implications for funding/granting agencies that are not conducive to the long-term trust and relationship building with stakeholders at the time of grant writing and conceptualisation of research that are essential for achieving co-production [112]. Funding duration is also limited to a few years, which works against developing long-term relationships with stakeholders. One solution could be that research funding agencies provide researchers with an opportunity to apply for seed grants to support the co-design of a full research proposal in collaboration with multiple stakeholders. There are initiatives in Canada, such as Coalitions Linking Action and Science for Prevention (CLASP) led by the Canadian Partnership Against Cancer, that provide allowances to researchers to develop relationships and coalition with stakeholders as a granting prerequisite [112].

We argue that stability and effectiveness of multi-stakeholder engagement depends strongly on trust, ongoing interactions and relationships, open communication, and knowledge sharing and co-learning. Our findings reinforce the primacy of trust in multi-stakeholder engagement research. Successful multi-stakeholder engagement hinges on trust, especially when partnership is of a voluntary nature. We argue that engagement and trust building in IKT research is typically voluntary and depends on a sense of commonality and reciprocity in win–win partnerships [113]. Stakeholders from disempowered groups are argued to have low levels of trust in multi-stakeholder partnerships [114], and ongoing interpersonal relationship-building is key to enhancing the trust of these groups [114, 115]. A win–win partnership with the realisation of non-zero-sum games between stakeholders based on mutual respect, mutual benefit and mutual trust are crucial to meaningful engagement of multiple stakeholders [116, 117]. For example, the co-framing of employment of PWDD that presents the business case and competitive advantage of employing a diverse workforce rather than a charitable perspective (implying it is a nice thing to do or to ‘help’), demonstrates a safe win–win space for collaboration for all stakeholders.

Co-learning in multi-stakeholder engagement is a win–win strategy as stakeholders learn from each other during the engagement process. This requires a shift from a static approach to learning, based on information acquisition, towards a greater emphasis on information distribution/exchange that leads to co-learning. Engagement provides opportunities for stakeholders to (1) share knowledge, (2) make tacit knowledge explicit, and (3) integrate explicit and tacit knowledge and shape it into usable knowledge [118]. Co-learning in IKT research addressing wicked problems means multiple stakeholders gain an improved understanding of the diversity of views on the problem and its policy solutions, which is critical for the success of multi-stakeholder collaborations [88]. As our engagement efforts matured over time, we developed more win–win and reciprocal relationships, which resulted in greater engagement from stakeholders. For example, greater engagement encouraged some employers from the first event to showcase their success stories with other stakeholders in our second event.

Finally, we argue that trust is not the only factor motivating or impeding engagement of stakeholders in IKT research. The accountability of stakeholders to engagement and its results, particularly in the case of PWDD and policy/decision-makers, is a key factor in successful IKT research [119]. In our co-production events (i.e. policy dialogues) we had limited representation from PWDD and policy/decision-makers, which implies that they were reluctant to participate in co-production, perhaps they did not know it was co-production given their past experiences. In the case of PWDD this could have been a reluctance to buy-in to the policy dialogue as a result of its structure or format. For policy or decision-makers, a potential fear of expectations to make policies in line with the knowledge co-produced could have been the case. However, as our results showed, PWDD were interested in being engaged in an alternate way, and policy/decision-makers were interested to hear and discuss about the research results and even utilise them. This, in our view, has implications for IKT research. As such, stakeholder dialogue events might not be the best model/strategy to engage particular stakeholder groups (PWDD and policy/decision-makers) in knowledge co-production. The lack of meaningful engagement by policy/decision-makers in the second event influences the impact of IKT research or co-produced knowledge on policy and practice [105]. As policy/decision-makers were not part of the participatory consensus-building process, there is potential for limited compliance with the results of co-production to improve policy and practice [37, 38, 120].

### Study limitations

A limitation of our research is that, although we sought stakeholders feedback following the two stakeholder dialogue events, we did not systematically evaluate their satisfaction with their role, their engagement, their expectations or their perspective on the engagement impact [18]. There are arguments that a lack of understanding about roles, responsibilities and expectations among stakeholders are major barriers to achieving successful and meaningful stakeholder engagement in research [121]. Although stakeholders disseminated research findings throughout their networks, a limitation of our engagement plan was a lack of stakeholders’ engagement in the dissemination of research findings through peer-reviewed as well as non-peer-reviewed publications and presentations as co-authors, which arguably enhances co-learning [122, 123].

## Conclusions

Although there is a growing emphasis on IKT research that entails active and ongoing collaboration between researchers and stakeholders throughout the entire research process to co-produce knowledge, little is known about best practices to meaningfully engage multiple stakeholders. Our experiences of engaging a wide range of stakeholders who hold different ideas and interests and come from different institutions to co-frame the wicked problem of employment for PWDD and co-produce policy solutions provided valuable lessons for other researchers and funding/granting agencies. In addition to providing adaptable engagement strategies, our paper contributes to discussions surrounding how IKT projects seeking effective and meaningful stakeholder engagement are planned and funded. Elaborating on our engagement strategies over a 2-year period, we provide recommendations in four areas: (1) call for further research to find optimal engagement strategies for policy/decision-makers, (2) call for documentation of successful engagement strategies, (3) investigating the impact of multi-stakeholder engagement, and (4) investigating representativeness of stakeholders participating in engagement. During our 2-year engagement activities, we observed a need for research on other methods/strategies of engagement that foster meaningful engagement of multiple stakeholders in IKT research, particularly policy/decision-makers, as our stakeholder policy dialogues seemed to be unsuccessful to effectively engage this stakeholder group. We call for documentation and reports of successful engagement strategies that other researchers have employed. Further research is also needed to study the impact of multi-stakeholder engagement. There is limited empirical evidence of the impact of knowledge co-produced through a true IKT (i.e. meaningful engagement of multiple stakeholders through the entire process of research) on policy and practice and we call for further research to fill this knowledge gap. Further research on IKT could also examine the stakeholders’ representativeness for engagement, or, in other words, how to ensure stakeholders engaged in IKT research are a true representative of their stakeholder group. This is particularly important for engaging PWDD and their families/caregivers, who are a heterogeneous population group.

## Supplementary information

**Additional file 1.** NGT steps to prioritise barriers to employment for persons with a developmental disability.

## Data Availability

In accordance with our approved research ethics protocol, the data generated during our 2-year engagement plan will not be publicly available in order to preserve participants anonymity.

## Abbreviations

AISH: Assured Income for the Severely Handicapped
PWDD: Persons with developmental disabilities
IKT: Integrated knowledge translation
CIHR: Canadian Institutes of Health Research
HR: Human resources
NGT: Nominal group technique
FG: Focus group
