# Supplementary material for: Stakeholders’ engagement in co-producing policy-relevant knowledge to facilitate employment for persons with developmental disabilities
Source: Health Res Policy Syst. 2020 Apr 17;18:39. doi: 10.1186/s12961-020-00548-2 (PMC7164207; doi:10.1186/s12961-020-00548-2)
Supplement: Supplementary file 1 — Additional file 1. NGT steps to prioritise barriers to employment for persons with a developmental disability. [file 12961_2020_548_MOESM1_ESM.docx]

Appendix 1: NGT steps to prioritize barriers to employment for persons with a developmental disability

| **Step** | **Activity** |
| --- | --- |
| **Step One:**  **Silent Generation** | The lead facilitator presented the key question: “what challenges do people with developmental disabilities face in entering the workforce and maintaining a meaningful employment?” and participants were asked to write down, individually, their ideas, as many they want, in silence. |
| **Step Two:**  **Round Robin** | Participants were asked, in turn, to share their ideas, which were recorded on flipcharts, visible to the entire group, by the table facilitators. Here each table facilitator went around the table and asked for one idea from one participant at a time and wrote them on a flipchart. Participants were not allowed to discuss, ask questions, or comments at this step. They were instructed to contribute only ideas that have not been mentioned by other participants or add a different perspective to what has already been mentioned. |
| **Step Three:**  **Clarification** | Participants discussed, clarified, and elaborated on collected ideas and determined their relative importance. |
| **Step Four: Categorizing** | Table facilitators with the help of participants organized the list of collected ideas into thematic categories by placing similar ones in a category. These were written on a new flip chart and were discussed and approved by participants. |
| **Step Five:**  **Ranking** | Each participant was asked to individually and anonymously prioritize the five most important barriers generated in the previous step and to rank order them in terms of importance. For each of the five selected barriers each participant chose between one and five points (their first preference five points and their fifth preference one point). The table facilitators combined all scores to establish a collective ranking for each table. To obtain the rating of the entire group, the lead facilitator collected the three top ranked barriers of each table and recorded them on a flip chart. The research team decided to identify the top three most important barriers for the entire group. Participants were then asked to individually and anonymously prioritize the three most important barriers and to rank order them in terms of importance. The lead facilitator combined all participants’ scores and announced the top three prioritized barriers. |
